# The influence of the introduction of national guidelines on preterm birth prevention practice: UK experience

**DOI:** 10.1111/1471-0528.15549

**Published:** 2018-12-28

**Authors:** A Care, L Ingleby, Z Alfirevic, A Sharp

**Affiliations:** ^1^ Harris‐Wellbeing Preterm Birth Research Group Centre for Women and Children's Health Research Liverpool Women's Hospital University of Liverpool Liverpool UK

**Keywords:** Preterm birth, short cervix, specialist antenatal clinic, transvaginal ultrasound

## Abstract

**Objective:**

To identify the current status of specialist preterm labour (PTL) clinics and identify changes in management trends over the last 5 years following release of the NICE preterm birth (PTB) guidance.

**Design:**

Postal Survey of Clinical Practice.

**Setting:**

UK.

**Population:**

All consultant‐led obstetric units.

**Methods:**

A questionnaire was sent by post to all 187 NHS consultant‐led obstetric units. Units with a specialist PTL clinic were asked to answer a further six questions defining their protocol for risk stratification and management.

**Main outcome measures:**

Current practice in specialist PTL clinics. Changes in treatment trends over 5 years.

**Results:**

Thirty‐three PTL prevention clinics were identified, with 73% running weekly. NHS staff (84%) have replaced university staff as the lead clinicians (from 69% in 2012 to 21% in 2017), suggesting this clinic has become increasingly integrated with standard care for women at the highest risk of PTB. There has been a large shift from nearly half of clinics offering cerclage as primary treatment for short cervix to offering more choice (30%) between at least two of cerclage, vaginal progesterone or pessary and combinations of primary treatments (18%), demonstrating more equipoise among clinicians regarding therapies for short cervix.

**Conclusions:**

Over 5 years, there has been a 44% increase in the number of specialist PTL clinics in the UK. Although there is a better consensus over the target high‐risk population, there is increasing heterogeneity among first‐line treatments for short cervix.

**Tweetable abstract:**

UK PTB prevention clinics have increased by 44% over 5 years, with increasing clinical equipoise to best Rx for short cervix.

## Tweetable abstract

UK PTB prevention clinics have increased by 44% over 5 years, with increasing clinical equipoise to best Rx for short cervix.

## Introduction

Implementation of strategies to prevent spontaneous preterm birth (sPTB) is increasingly standardized, as several recent RCTs and meta‐analyses have shown that treatment for short cervical length detected by transvaginal ultrasound scan may reduce the risk of PTB.^1^, ^2^, ^3^, ^4^, ^5^, ^6^, ^7^, ^8^ A systematic review of clinical practice guidelines (CPGs) for the prevention and management of sPTB worldwide found there were nine areas of consensus, including cervical length screening for high‐risk women, but no consensus on treatment. There remain other areas of disagreement among clinical experts and guideline committees, with some recommended practices deemed ineffective based on current evidence and several contradictory recommendations.^9^

Information on the impact of PTB guidelines on clinical management has not been available to date. It is unclear whether these global variations in recommended practice are also seen within nations or guidelines are strictly followed once implemented.

Considerable variation in management was demonstrated by our previous survey of UK specialist preterm labour (PTL) clinics in 23 clinics established in the UK, performed in 2012.^10^ No consensus was available on the indications for referral, gestation of screening, definitions of the ‘at risk’ population or cervical length treatment thresholds. However, we concluded that antenatal PTL clinics were ‘here to stay’ as part of a growing trend to have specialist antenatal clinics. At the time of the previous survey, there had been a lack of formal guidance from health regulatory bodies on PTB prevention and the role of specialist clinics.

In 2015, the National Institute for Clinical Excellence (NICE) in the UK published a CPG for the management of PTL and birth.^11^ Although specialist clinics were not formally mentioned, this guideline clearly identifies 16^+0^ to 24^+0^ weeks as the gestational age suitable for detection of a short cervix, defined as <25 mm on transvaginal ultrasound scan. Prophylactic vaginal progesterone or cervical cerclage is advised for women with a history of spontaneous preterm birth or mid‐trimester loss between 16^+0^ and 34^+0^ weeks with a short cervix. Additionally, increased guidance is provided for clinicians on the diagnosis and management of preterm labour for women presenting with symptoms. No mention is made of the use of the Arabin cervical pessary.

We performed a repeat survey 5 years after the original survey to identify whether current UK management of prevention of PTL has become increasingly homogenised following the issue of the NICE guidelines, and to identify current practice of diagnosis and management of women with threatened PTL in UK hospitals.

## Methods

A postal survey (Supporting Information Appendix S1) comprising 21 multiple‐choice questions addressing recommendations made by the NICE preterm birth guidelines 2016 was sent to 187 consultant‐led NHS hospitals in the UK (England, Wales, Scotland, Northern Ireland, Channel Islands, and Isle of Man). The first round was sent in March 2017, followed by a second round in July 2017 to those who had not responded to the initial invitation. Questions focused on indications for referral to preterm birth prevention clinics, if available, and management of asymptomatic women at risk of spontaneous preterm birth. Additionally, this questionnaire asked about assessment and management of women attending the unit with threatened preterm labour, irrespective of preterm birth prevention clinic status. Respondents were given the opportunity to include written responses when predesigned response options were not applicable. Respondents could reply by post using the pre‐addressed envelope provided or via email. If clarity was required on an answer, the email address provided by the staff member completing the questionnaire was contacted to clarify. Comparison of results was made with our previous UK preterm birth survey conducted in 2012,^9^ which only focused on the role of preterm birth prevention clinics to assess the changes in service provision over the last 5 years. In this round, the role of management of threatened preterm labour was also included to assess the level of heterogeneity in management throughout the UK following release of the NICE guidelines on preterm birth. There was no patient involvement in the development of this research, and the study was unfunded.

## Results

Since 2013, 11 units previously included in the survey no longer provide obstetric services or have merged, leaving 187 hospital trusts identified for inclusion. A postal survey was performed in two rounds, March and July 2017, achieving an overall response rate of 106/187 (57%). In all, 33/106 (31%) of units had specialist PTL clinics: 30 clinics in England, two in Scotland, and one in NI.

The majority of clinics run weekly and are led by NHS obstetric consultants (Table 1); there was a noticeable reduction in university staff leading the clinic, from 69 to 21% over 5 years in favour of NHS staff (84%). Indications for referral to specialist preterm labour clinics are shown in Table 2 and include: previous spontaneous preterm labour (100%), two large loop excisions of the transformation zone (LLETZ; 100%) and single knife cone biopsy (100%) as universal antenatal indications for referral to clinic with ‘previous premature rupture of membranes (PPROM’; 91%), and ‘recurrent second trimester miscarriages’ (91%) referral criteria in almost all clinics. The most common gestation of previous sPTB or PPROM triggering referral remains at less than 34 weeks (65%). In all, 88% of clinics manage incidental findings of a short cervical length in pregnancy, and four clinics manage follow up for women with threatened preterm labour combining populations of asymptomatic and symptomatic women at risk of spontaneous PTL. An initial appointment following referral is most common between 12 and 14 weeks of pregnancy (84%).

**Table 1:** Frequency and staffing of specialist preterm labour clinics. Values are given as *n* (%)

	2012	2017
Frequency of preterm labour clinic	NR	*n* = 33 (100%)
Twice weekly	NR	2 (6)
Weekly	NR	24 (73)
Fortnightly	NR	7 (21)
Monthly	NR	0

Staffing of preterm labour clinic		
Lead clinician – NHS staff	7 (30)	28 (84)
Lead clinician – University staff	16 (69)	7 (21)
Designated midwife	11 (55)	17 (52)
Not stated	0	1 (3)

Cervical length assessment operator		
Obstetric consultant	17 (77)	28 (85)
Obstetric trainee	8 (36)	14 (42)
Staff grade	2 (9)	3 (9)
Research/clinical fellow	6 (27)	7 (21)
Midwife	3 (14)	5 (15)
Ultrasonographer	9 (41)	10 (30)

n, number of preterm labour (PTL) clinics; NR, Not recorded.

**Table 2:** Referral and management structure of specialist preterm labour (PTL) clinics in UK. Values are given as *n* (%)

Indication for referral to PTL clinic (non‐exclusive)	2012 *n* = 21	2017 *n* = 32^a^	Gestation of previous PTB	2012 *n* = 21	2017 *n* = 31^b^
Previous spontaneous PTB	21 (100)	32 (100)	<37 weeks	1 (14)	4 (13)
Previous PPROM	20 (95)	29 (91)	<35 weeks	1 (5)	1 (3)
1 × LLETZ	11 (52)	15 (47)	<34 weeks	10 (48)	20 (65)
2 × LLETZ	20 (95)	32 (100)	<32 weeks	5 (24)	4 (13)
Cone biopsy	20 (95)	32 (100)	<28 weeks	2 (10)	1 (3)
Uterine anomalies	19 (90)	24 (75)	Other	0	1 (3)
Recurrent first trimester miscarriage	1 (5)	5 (16)			
Recurrent second trimester miscarriage	20 (95)	29 (91)	Gestation of previous PPROM		
Threatened PTL	NR	4 (13)	<37 weeks	NR	5 (16)
Incidental CL finding	NR	28 (88)	<34 weeks	NR	17 (55)
Other	0	5 (16)	<32 weeks	NR	4 (13)
			<28 weeks	NR	2 (6)
			Other	NR	3 (10)

Initial clinic appointment (non‐exclusive)	2012 *n* = 20	2017 *n* = 32^a^			
<12 weeks	11 (55)	3 (9)			
12–14 weeks	3 (15)	12 (38)			
15–16 weeks	2 (10)	16 (50)			
>16 weeks	3 (15)	1 (3)			
As soon as referred	1 (5)	0			

CL, cervical length; LLETZ, large loop excision of transformation zone; n, number of preterm labour (PTL) clinics; PPROM, preterm prelabour rupture of membranes.

a One unit did not respond.

b Two units did not respond.

Table 3 shows details of prophylactic treatment, cervical length assessment, and treatment choice for short cervix. A short cervical length is classified as <25 mm in 55% of PTB prevention clinics and 63% of units that do not have a clinic; however, centile charts appropriate for gestational age (27%), <15 mm cervical length (3%), and the use of the QUIPP app (12%) are increasingly also used. The most popular treatment choice for short cervix is cervical suture (30%). An equally popular alternative to cerclage is to offer women a choice of multiple therapies (30%) with a combination of vaginal progesterone and cervical cerclage being the most common. Use of vaginal progesterone remained consistent across 5 years with 18% of units still using it as first‐line treatment of a short cervix. Vaginal progesterone for prophylaxis based on history alone is generally avoided, with only 6% of units prescribing it without evidence of a short cervix.

**Table 3:** Comparison of treatment cut‐offs for asymptomatic population and primary treatment choices over 5 years and between PTB clinics and units that do not have a preterm birth clinic. Values are given as *n* (%)

	2012 PTL Clinic *n* = 22	2017 PTL Clinic *n* = 33	2017 No PTL Clinic *n* = 71^a^
**Cervical length at treatment**			
<25 mm	13 (59)	18 (55)	45 (63)
<15 mm	2 (9)	1 (3)	6 (8)
Centile Chart cut‐off	3 (14)	5 (15)	3 (4)
Centile Chart cut‐off AND/OR <25 mm	0 (0)	4 (12)	2 (3)
QUIPP app	0 (0)	4 (12)	2 (3)
Other CL cut‐offs	4 (18)	1 (3)	8 (11)
Individualised based on history/clinical change/clinician	0	0	5 (7)
**Primary treatment choice**			
Cervical cerclage	10 (45)	10 (30)	41 (58)
Vaginal progesterone	4 (18)	6 (18)	11 (15)
IM Progesterone	0	0	0
Cervical pessary	1 (4)	1 (3)	0
Combination therapy	5 (22)	6 (18)	3 (4)
Multiple first‐line treatment options	2 (9)	10 (30)	16 (23)

a Two units do not perform TVU CL assessment of cervical length.

When primary treatment for short cervix is considered to be failing, cervical cerclage (30%) was the most popular secondary treatment. If cerclage was already a first‐line treatment, vaginal progesterone was the most popular second line. However, 23 clinics did not specify a secondary choice.

In units that do not have dedicated preterm labour clinics, ‘combination therapy’ is less common (4%) and cervical cerclage is overwhelmingly the first‐line treatment choice with over half of all women with short cervix being offered this treatment (Table 3).

### Managing threatened preterm labour

#### Diagnosis

A total of 106 units reported their management of women presenting with symptoms of preterm labour. Cervical measurement with transvaginal ultrasonic scanning (TVUSS; 58%) remains the most frequently used test, but only 10% of units perform this assessment alone. Nearly, all units use ultrasound in combination with another bedside diagnostic test (Table 4). The phosphorylated form of insulin‐like growth factor‐binding protein‐1 (51%), fetal fibronectin (fFN; 39%), and placental alpha macroglobulin‐1 (PAMG‐1; 3%) is currently used either in isolation or combined with other diagnostic biomarkers, or as an adjunct to cervical length scanning; only two hospitals use clinical assessment alone without any diagnostic test adjunct.

**Table 4:** Assessment of symptomatic women at risk of preterm labour and treatment choice for starting tocolysis in threatened preterm labour. Values are given as *n* (%)

Assessment tool for symptomatic women	(Total response *n* = 106)		
Clinical assessment only	1 (1)		
Actim Partus alone	11 (10)		
**FFN alone**	31 (29)		
+ Actim Partus	1 (1)		
**CL TVUUS**	62 (59)		
+ alone	11 (10)		
+ Actim Partus	40 (38)		
+ FFN	5 (5)		
+ FFN + Actim Partus	3 (3)		
+ Partosure	2 (2)		
+ FFN (Qualitative) + Partosure	1 (1)		

First choice tocolytic (non‐exclusive)	(Total response *n* = 106)		
Nifedipine	75 (71)		
Atosiban	29 (27)		
Progesterone	2 (1)		
GTN	1 (1)		
Indomethacin	1 (1)		
Do not use tocolysis	2 (2)		

Gestation fortocolysis use	(Total response *n* = 106)		(Total response *n* = 106)
Earliest gestation (weeks)		Latest gestation (weeks)	
16	1 (1)	24	1 (1)
20	1 (1)	32	4 (4)
22	5 (5)	33	3 (3)
23	31 (29)	34	72 (68)
24	59 (56)	35	9 (8)
25	1 (1)	36	6 (6)
26	4 (4)	<37	1 (1)
Depending upon history	1 (1)	Depending upon history	2 (2)
Not given	0	Not given	5 (5)
Do not use tocolysis	3 (3)	Do not use tocolysis	3 (3)

Offer rescue cerclage (if no signs of infection)	(Total response *n* = 106)		
Yes	58 (55)		
No	7 (6)		
Depends on gestation	41 (39)		

#### Tocolysis

Nifedipine (71%) and atosiban (27%) are the most popular first choice tocolytics (Table 4). The earliest gestation that tocolysis was offered ranged from 16 to 26 weeks and the upper gestation of use ranged from 26 weeks to less than 37 weeks’ gestation (*n* = 104, 98%). Two trusts did not use tocolysis (2%).

#### Rescue cerclage

In asymptomatic women with exposed or prolapsed membranes and no sign of infection, 99 units (94%) would offer rescue cerclage in some circumstances (Table 4). Forty‐one units (39%) indicated that the gestational age was an important factor in offering rescue cerclage. Seven units (6%) do not offer rescue cerclage due to a combination of: risk of infection (*n* = 3, 3%), limited experience (*n* = 2, 2%), lack of scientific evidence (*n* = 8, 8%), and clinical ineffectiveness (*n* = 2, 2%).

## Discussion

### Main findings

This survey was a follow up to a structured assessment carried out in 2012/2013 of identification and management of women at risk of PTL in the UK. Over 5 years, there has been an increase in specialist PTL clinics, possibly reflecting a growing focus on these services following publication of national guidance and increased patient demand. Unsurprisingly, the greatest proportion of specialist PTL clinics are located within larger maternity units, perhaps reflecting bigger demand, as well as the availability of clinicians and resources to support these services. Previously, all identified specialist PTL services were located only in England; the services are now in devolved nations, making coverage more consistent with UK population demands. The reduction in university staff from 69 to 12% demonstrates these clinics may no longer be predominantly research‐focused and have transitioned into standard clinical care.

There is certainly greater consensus among the management of PTL clinics compared with 2012. There was a lack of consensus over cervical length measurement deemed significant to initiate treatment. NICE defined short cervix in an asymptomatic population as a transvaginal cervical length of <25 mm, and our survey shows that 77% of units have adopted these new guideline recommendations.

### Strengths and limitations

The response rate for this survey was 57% and may not have achieved a complete picture of current UK practice. We did not include questions on management or screening of multiples for PTL. This was a pragmatic decision to ensure questionnaires were not overly time‐consuming and to try to maintain good response rates. Improving response rates is unlikely to change the overall conclusion of this study. The lowest response was from hospitals with <2500 births per annum (*n* = 15). Most units in this group may only manage low‐risk maternities and are likely to refer high‐risk patients to larger units. Therefore, we feel that we achieved adequate coverage across various size units, particularly those actively managing preterm births.

### Interpretation

Most women attending for their first appointment at clinic are at 12–14 weeks’ gestation, and <34 weeks is emerging as the most popular cut‐off gestation to identify high‐risk women based on a previous PTL and PPROM, which has increased from 48% since 2013 to 65% today. In contrast to these findings, there is increasing divergence over primary treatments used for short cervix. Consistent with the current equipoise in research literature, clinics are increasingly offering patients the choice of combined therapies, in the hope that they will work on mechanistically different biological pathways. As an example, the most popular treatment choice for short cervix is cervical suture (30%) but this is compared with nearly half (45%) of clinics using cerclage 5 years previously. This change may have been partly related to National Institute of Health Research **(**NIHR)**‐**funded clinical trials such as RECAP (EudraCT no. 2014‐003112‐36) and SUPPORT (EudraCT no. 2015‐000456‐15) encouraging randomisation between all three treatments which have been recruiting since the last questionnaire study was performed.^12^ Assuming that there is no clinically meaningful difference between these three treatments and all are subsequently proven to provide benefit, our next challenge will be to identify whether combination therapy is of added value, as this practice has started to increase. We will need more adaptive randomised trial designs as we try to power studies to demonstrate ever smaller clinical effect sizes in a relatively small population at high risk.

Cervical cerclage remains the most popular first‐line treatment choice, particularly in units without specialist preterm birth prevention services. This is likely to be the consequence of NICE guidance in combination with high‐profile negative trials of vaginal progesterone^13^ and the Arabin pessary^14^ published in recent years.

We hope to see more homogeneity among PTB clinics following increased communication between specialist clinics at a national level through the UK Preterm Birth Clinical Network with increased support for hospitals developing new clinics.^15^ Changes in primary treatment will almost certainly come from further research showing definitively positive or negative results for existing or new treatments. Specialist PTL clinics currently provide an environment to set‐up and encourage participation in ongoing studies of women most at risk of PTL which will remain key for coordinated research efforts to identify pathophysiology and treatment of spontaneous PTL, particularly in light of the UK health secretaries’ pledge to reduce PTB from 8 to 6% by 2025.^16^

Transvaginal ultrasound was the most commonly used tool both on its own and in combination with other therapies. Interestingly, only 16% of the health professionals performing transvaginal ultrasound in units without specialist preterm labour clinics are speciality trainees. This figure perhaps may only reflect permanent clinic staff and may not reflect training of speciality trainees rotating in these clinics sporadically. However, it is important to mention that specialist PTB clinics provide excellent educational environments for training of cervical length scanning supervised by experienced clinicians in a non‐acute environment. Although TV cervical length measurement is not considered to be a requirement of Royal College of Obstetrics & Gynaecology (RCOG) basic, intermediate or advanced ultrasound training, this skill has been identified as the gold standard of threatened PTL diagnostic assessment. We suggest that more trainees should be encouraged to acquire and use this ultrasound skill, as they are more likely to look after women with acute symptoms of PTL which may include decisions to administer steroids, tocolysis, and magnesium sulphate.

Despite the NICE guidelines advocating the fetal fibronectin bedside test when TVUSS is not available,^11^ is the most popular rapid bedside test used in practice.

Over 70% of trusts follow the NICE recommendation of calcium channel blocker nifedipine as the first choice tocolytic. Atosiban was also reported in 20% of the remaining responses. Although similar efficacy levels have been reported in both direct and indirect comparative studies,^17^, ^18^ a decision analysis and network meta‐analysis both found that calcium channel blockers would be a preferred first‐line tocolytic with regard to several important outcomes including 48 hours’ delay in delivery, respiratory distress syndrome, neonatal mortality, and maternal side effects.^19^

On discovery of premature cervical dilation, often with exposed or prolapsing membranes, a rescue cerclage may be considered. Most units would offer a rescue cerclage if no obvious infection was detected in at least some clinical circumstances (94%); however, the questionnaire did not explore eligibility criteria for this procedure per unit. There is, however, a clear need for more robust data on maternal and fetal outcomes following rescue cerclage, which poses a significant challenge. A recent NIHR‐commissioned call for a randomised controlled trial (RCT) of rescue cerclage versus expectant management may be able to provide this much‐needed data.

## Conclusion

Over the last 5 years, there has been an increase in the number and geographical spread of specialist preterm birth clinics in the UK. Although variation in practice remains, there appears to be increasing consensus in cut‐offs for cervical length treatment, referral criteria to clinic, and the gestation at first appointment. Interestingly, there is increasing equipoise regarding primary treatments for short cervix, with more choice of first‐line treatments and combination treatments offered to women.

The most favoured diagnostic tool for symptomatic threatened preterm labour is cervical length measurement in combination with testing (38%), but there is still large variation in UK methods of diagnosis of preterm labour.

### Disclosure of interests

None declared. Completed disclosure of interest forms is available to view online as supporting information.

### Contribution to authorship

AS conceived the idea for the manuscript. AC drafted this article, which was subsequently critically reviewed and revised by LI, AS, and ZA. AC, LI, and AS designed the questionnaires and LI arranged for questionnaires to be sent to all obstetric units. LI collated the data and AC, LI, and AS performed analysis and produced the data tables. AS and ZA provided data from 2012 for comparison.

### Details of ethics approval

Ethical approval was not required as no patient identifiable information was used.

### Funding

No funding was required for this study.

## Supporting information


**Appendix S1.** Survey of practice in preterm labour clinics in the UK.
