# Supplementary material for: The influence of the introduction of national guidelines on preterm birth prevention practice: UK experience
Source: BJOG. 2018 Dec 28;126(6):763–9. doi: 10.1111/1471-0528.15549 (PMC6590292; doi:10.1111/1471-0528.15549)
Supplement: Supplementary file 1 — Appendix S1. Survey of practice in preterm labour clinics in the UK. [file BJO-126-763-s001.pdf]

UNIVERSITY OF LIVERPOOL

Liverpool Women's NHS
NHS Foundation Trust

Appendix S1. Survey of Practice in Preterm Labour Clinics in the United Kingdom

Background

Our previous survey of practice (Sharp and Alfrievic, BJOG 2013) highlighted a wide variation in the management of high-risk asymptomatic women with a short cervix length. Since then there has been an increasing desire by some clinicians to provide specialised care for women at risk of preterm birth and further changes recently advocated by NICE (NOV 2015). In light of this we wish to review how the practise and provision of care for women at risk of preterm birth has changed over time.

Aim

To identify the current state of preterm labour special services within the UK

Please tick the response that best represents your department's policy.

If answering other, please specify how your management differs from the written responses.

Questions

1. How many deliveries does your unit have per year? ______________________

2. Do you have a dedicated pre term labour (PTL) clinic?
   YES •
   NO •

If you don't have a dedicated PTL clinic please go to question 9

3. How frequent is this clinic?
   a. Weekly •
   b. Fortnightly •
   c. Monthly •
   d. Other __________

4. Who is the clinical lead for this clinic?
   a. NHS consultant (Principally Obstetrics) •
   b. NHS consultant (Principally Gynaecology) •
   c. Subspecialist •
   d. University Staff Clinician •
   e. Specialty Trainee Doctor •
   f. Midwife •
   g. Other (please state) ____

5. Do you see patients with a history of – (tick all that apply)
   a. Previous Spontaneous Preterm Birth •
   b. Previous PPROM •

c. Cervical surgery – Single LLETZ •
d. Cervical surgery – Multiple LLETZ •
e. Cervical surgery – Cone Biopsy •
f. Uterine anomalies •
g. Recurrent 1st trimester miscarriage •
   i. How many? ______________________________________
h. Recurrent 2nd trimester miscarriage •
   i. How many? _______________________________________
i. Following episode of threatened preterm labour •
j. Incidental finding of short cervix without PTB history •
k. Other (not listed) •
   Please describe ____

6. What gestation of previous spontaneous preterm labour (PTL) do you consider to be an indication for referral to the PTL clinic or consultant assessment?
   a. Delivery <37 weeks •
   b. Delivery <34 weeks •
   c. Delivery <32 weeks •
   d. Delivery <28 weeks •
   e. Other ________________

7. What gestation of preterm prelabour rupture of membranes (PPROM) do you consider to be an indication for referral to the PTL clinic or consultant assessment?
   a. Delivery <37 weeks •
   b. Delivery <34 weeks •
   c. Delivery <32 weeks •
   d. Delivery <28 weeks •
   e. Other ________________

8. At what gestation do you see patients for their first appointment?

   _______________________ Weeks

9. For asymptomatic women at risk of preterm labour, do you offer prophylactic vaginal cerclage on history alone without surveillance with ultrasound?
   YES •
   NO •
   SOME (please describe) ____
   ____

10. For asymptomatic women at risk of preterm labour, do you offer prophylactic vaginal progesterone on history alone without surveillance with ultrasound?
    YES •
    NO •
    SOME (please describe) ____
    ____

11. Do you perform transvaginal ultrasound assessment of cervical length?
    YES •
    NO •

12. Who performs this scan? (Please circle all that apply)
    a. Consultant •
    b. Specialty Trainee •
    c. Non-training grade clinical staff •
    d. Research Staff •
    e. Midwife •
    f. Ultrasonographer •
    g. Other ____

13. At what cervical length would you initiate treatment in an asymptomatic high-risk woman?
    a. ________________________ mm (Please provide a value)
    b. Based upon centile charts from cervical normogram •
    c. Based upon cervical length and fetal fibronectin level (e.g. QUIPP app) •
    d. Other __________________

14. What is your preferred primary treatment for short cervical length?
    a. Vaginal progesterone •
    b. Cervical cerclage (Braided suture) •
    c. Cervical cerclage (Monofilament suture) •
    d. Vaginal pessary (such as Arabin) •
    e. IM progesterone •
    f. Combination treatment (please state):_________________________________
    g. Other ____

15. If primary treatment for short cervical fails, what is your choice of secondary treatment?
    Please State ____

16. How do you assess symptomatic women at risk of preterm labour? (Please circle all that apply)
    a. Cervical Length transvaginal ultrasound •
    b. Fetal Fibronectin (Qualitative) •
    c. Fetal Fibronectin (Quantitative) •
    d. Actim Partus •
    e. Other ___________________

17. If starting tocolysis for threatened preterm birth, what is the medication of first choice in your unit?
    a. Nifedipine •
    b. Atosiban (tractocile) •
    c. Indomethacin •
    d. Progesterone •
    e. Other ________________________

18. At what gestation do you offer tocolysis?

    Earliest Gestation: ___________ weeks

    Latest Gestation: ___________ weeks

19. Do you offer 'rescue' cerclage for prolapsed membranes in asymptomatic women with no sign of infection?

    YES •

    NO •

    It depends on gestation •

20. If you don't offer 'rescue' cervical cerclage, why not?
    a. Lack of scientific evidence •
    b. It doesn't make a difference •
    c. Risk of infection •
    d. Limited experience •
    e. Other _____________________________________

21. Would you wish to be contacted about future studies of preterm birth or 'rescue' cervical cerclage?

    YES •

    NO •

Name __________________________________

Email __________________________________

Thank you for your time in completing this questionnaire
